# Genome sequencing and the diagnosis of novel coronavirus (SARS-COV-2) in Africa: how far are we?

**DOI:** 10.11604/pamj.2020.36.80.23723

**Published:** 2020-06-09

**Authors:** Muki Shey, Joseph Chukwudi Okeibunor, Ali Ahmed Yahaya, Belinda Louise Herring, Oyewale Tomori, Sheick Omar Coulibaly, Hieronyma Nelisiwe Gumede-Moeletsi, Jason Mathiu Mwenda, Zabulon Yoti, Charles Shey Wiysonge, Ambrose Otau Talisuna

**Affiliations:** 1 Welcome Centre for Infectious Disease Research in Africa (CIDRI-Africa), Institute of Infectious Disease and Molecular Medicine (IDM), University of Cape Town, Cape Town, South Africa,; 2 Department of Medicine, University of Cape Town, Cape Town, South Africa,; 3 World Health Organization (WHO), Africa Office, Brazzaville, Congo,; 4 Cochrane Centre, South African Medical Research Council, Cape Town, South Africa,; 5 School of Public Health and Family Medicine, University of Cape Town, Cape Town, South Africa

**Keywords:** RT-PCR, SARS-COV-2, COVID-19, serology test, antibody test, rapid diagnostic test (RDT), genome sequencing

## Abstract

The coronavirus disease (COVID-19) caused by the novel severe acute respiratory syndrome coronavirus 2 (SARS-COV-2) has become a pandemic. There is currently no vaccine or effective treatment for COVID-19. Early diagnosis and management is key to favourable outcomes. In order to prevent more widespread transmission of the virus, rapid detection and isolation of confirmed cases is of utmost importance. Real time reverse transcriptase polymerase chain reaction (RT-PCR) is currently the “gold standard” for the detection of SARS-COV-2. There are several challenges associated with this test from sample collection to processing and the longer turnaround time for the results to be available. More rapid and faster diagnostic tests that may produce results within minutes to a few hours will be instrumental in controlling the disease. Serological tests that detect specific antibodies to the virus may be such options. In this review, we extensively searched for studies that compared RT-PCR with serological tests for the diagnosis of COVID-19. We extracted the data from the various selected studies that compared the different tests and summarised the available evidence to determine which test is more appropriate especially in Africa. We also reviewed the current evidence and the challenges for the genome sequencing of SARS-COV-2 in Africa. Finally, we discuss the relevance of the different diagnostic tests and the importance of genome sequencing in identifying potential therapeutic options for the control of COVID-19 in Africa.

## Introduction

Since being detected in Wuhan, China, in December 2019, the novel severe acute respiratory syndrome coronavirus 2 (SARS-COV-2) has spread to 216 countries, areas or territories worldwide including 54 African countries where more than 95,000 cases have been confirmed with over 2,950 deaths and 34,000 recoveries [1]. SARS-COV-2 is the etiologic agent for the coronavirus disease (COVID-19), which is now a pandemic. Real time reverse transcription polymerase chain reaction (RT-PCR) is currently the test for the detection of SARS-COV-2 in human samples derived from the lower or upper respiratory tract [2]. Positive tests using blood and anal swabs have also been reported [3,4]. Swabs or fluid is used for the isolation of genetic material, RNA, which is reversed transcribed to cDNA and amplified in the presence of specific reagents (such as primers, probes), if the virus was present in the clinical samples. In the absence of the virus, no viral RNA will be present and consequently no amplification.

This test requires trained personnel for good sample collection and extraction of the RNA to avoid false negative results (or false positive results if contamination occurs) [5]. Appropriate laboratory infrastructure including biosafety cabinets, personal protective equipment (PPE) and instruments for the extraction, running and analysis of the results are also required [6]. RT-PCR is currently the “gold standard” for the detection of active infection. However, clinical indicators should also be taken into consideration for the diagnosis, treatment, management and isolation of patients [5]. The average turnaround time for RT-PCR diagnostic test may range from one to several days, in resource-limited settings. This test may be relevant for hospitalised patients but can be a challenge for mass screening or in cases where results are needed immediately for treatment and isolation of infected individuals. More rapid and cheaper detection tests that do not require highly trained personnel and complex infrastructure are needed to scale up testing especially in less-resourced environments. Serological tests that detect specific antibodies against the virus may offer such alternatives, but they are also associated with some challenges [6-8].

Since the beginning of the SARS-COV-2 outbreak, the World Health Organisation (WHO) has provided training to atleast 44 African countries who now have the capacity to perform RT-PCR testing to detect the virus [1]. Africa Centre for Disease Control and Prevention (Africa CDC) is also instrumental in supporting member states with reagents and equipment to facilitate testing for SARS-COV-2 [9]. In this review, we aimed to evaluate studies comparing laboratory testing by RT-PCR to other detection tests, mainly serology tests for antibodies against SARS-COV-2 (including the rapid diagnostic tests, RDTs) and their relevance in the fight against COVID-19 in Africa. We also summarise evidence for the SARS-COV-2 genome sequencing in Africa and the importance in the context of global data.

## Methods

We performed a search of peer-reviewed published articles indexed mainly on PubMed (28^th^ April 2020) for the diagnosis of SARS-COV-2 (Table 1). We also searched MedRxiv and BioRxiv for publicly available articles that may not yet have been peer-reviewed. Reference lists of published articles (including reviews) and documents on databases such as WHO, CDC and GISAID were also scanned for potential articles.

**Table 1:** search terms and number of studies identified

	Search terms	Hits
1	″SARS-CoV-2 RT-PCR″ or ″SARS-CoV-2 AND RT-PCR″	143
2	″SARS-CoV-2 antibody″ or ″SARS-CoV-2 antibody″	140
3	″2019-nCoV RT-PCR″ or ″2019-nCoV RT-PCR″	115
4	″2019-nCoV antibody″ or ″2019-nCoV antibody″	93

The hits in #2, #3, #4 were all mostly subsets of #1

## Current status of knowledge

We went through the search result articles and selected either the primary research articles or meta-analyses of articles that compared two or more tests for the detection of SARS-COV-2. Normal review articles or personal communication or articles reporting only treatment strategies (without a virus detection component) were not included in the summary, but some critical ones were referenced where necessary. Only articles reporting data (complete or partial) in English were selected. We categorized articles that compared RT-PCR and any antibody test (26 articles) (Table 2) [4,10-34], or articles that compared RT-PCR with any test that was described as “rapid” or “point-of-care” (8 articles) [35-42], for the detection of SARS-COV-2 (Table 2). The 26 articles comparing RT-PCR with serology test were geographically distributed as follows: Mainland China (20), Finland (1), Netherlands (1), France (1), Peru (1), Germany (1) and Hong Kong (1). The 8 articles comparing RT-PCR and rapid tests were from Mainland China (4), Italy (1), Germany (1), Brazil (1; meta-analysis) and Taiwan (1). A further 4 articles were found that describe the detection of neutralizing antibodies against SARS-COV-2 with no direct comparison with RT-PCR, mainly for therapeutic purposes and not discussed in this review. These were from Mainland China (2), USA (1) and Singapore (1) [43-46].

**RT-PCR and serology antibody tests to detect SARS-COV-2:** RT-PCR was used as the gold standard for detecting SARS-COV-2 and the antibody tests were compared to the RT-PCR test. All studies evaluated either immunoglobulin M (IgM) or IgG or both that are specific for SARS-COV-2. There was a wide variation between studies in the number of participants included or those who underwent molecular (and serology) testing for SARS-COV-2, ranging from 1 participant (five case reports) to 951 participants (though not all had both tests performed) (Table 2). The agreement between the RT-PCR and the antibody detection tests ranged from 100% to below 20%. The antibodies against SARS-COV-2 were detected from as early as 2 days post onset of symptoms to as late as 7 weeks or more post onset of symptoms. With a few exceptions that used plasma alone, most studies evaluated the antibodies using serum (sometime with plasma and whole blood as well) that was collected from participants at various times from admission to several weeks post recovery or discharge from hospitals (for hospitalized patients). The current wide range in the detection period (time to seroconversion) for the antibodies make them less useful as diagnostic tests for acute infection, but still very useful as supporting tests in determining previous infection (whether still an active infection or not) with SARS-COV-2. Further development and (independent) validation of these antibody tests is required. The CDC has designed and validated a serologic test which is currently being used to guide the response to the COVID-19 pandemic in the United States and they are also testing several commercially available serologic tests and results are expected soon [7].

Even though these were case reports and prone to reporting bias, two studies (1 participant each) reported data on COVID-19 in individuals co-infected with HIV (and one with HCV) [12,25]. Both studies reported delayed seroconversion and antibody detection, 4-7 weeks from onset of symptoms. One study reported persistent negative RT-PCR results [25], while the second reported one positive RT-PCR out of 4 serial tests [12]. If these are confirmed in larger powered studies, it would suggest antibody testing might be more relevant in the diagnosis of COVID-19 in HIV-infected participants. Currently no other studies have reported evaluation of the diagnosis of SARS-COV-2 in HIV-infected patients, especially those with advanced disease who are usually at high risk of developing other infectious diseases and complications [47,48]. One study evaluated diagnosis of SARS-COV-2 in a pregnant woman [17]. The woman tested positive for RT-PCR and antibodies and 16 hours after giving birth (by caesarian section), the newborn also tested positive for SARS-COV-2 by RT-PCR but did not have detectable antibodies. If confirmed in larger powered studies, this may have implications for vertical transmission of SARS-COV-2.

**Table 2:** studies comparing the use of molecular and serology assays for the detection of SARS-CoV-2 with key findings from each study

Article	Location (N)	Summary of key findings
Wang *et al*.	China (1)	HIV+ with low CD4 count (34cells/mL), 1 RT-PCR+ test (out of 4) and had detectable IgM but not IgG. IgM was detected 2 months after onset of symptoms
*Lou *et al*.	China (80)	All 80 patients were RT-PCR+ and 79/80 had detectable antibodies. 75/80 had detectable IgM or IgG. Seroconversion time was 9-12 days. For a subset of patients with known time of exposure, seroconversion was 15-20days
Xie *et al*.	China (56)	16 were RT-PCR+. Of the 16 RT-PCR positives, 1 tested negative for IgM antibodies by ELISA (all IgG+), while of the 40 RT-PCR negatives, 34 tested positive for the IgM antibody. IgG was positive in all 56 patients. Antibody detected from day 7 (from when serum samples collection started).
Qu *et al*.	China (41)	All 41 were RT-PCR+.40/41 IgG+ and 36/41 IgM+. Median conversion time. IgG 11 (8-16) days and IgM 14 (8-28) days
Yang *et al*.	China (1)	Patient was RT-PCR+. Antibody (IgG) detectable at day 40
Yongchen *et al*.	China (21)	21 tested positive for RT-PCR with varied disease severity.17/21 had detectable antibodies between week 1 to week 6.
Xiang *et al*.	China (109)	66 (RT-PCR+) patients underwent both tests. 51/66 had detectable antibodies. Seroconversion was between 4-12 days
Alzamora *et al*.	Peru (1)	A pregnant lady confirmed to be RT-PCR+ 15 days from symptoms onset. Also had detectable antibodies at day 15. Gave birth to infant who tested RT-PCR positive with no detectable antibodies 16hours after birth.
*Lin *et al*.	China (159)	Only 79 underwent molecular testing and were all RT-PCR+.72/79 had detectable total IgM/IgG+. 65/79 had detectable IgM+. 65/79 had detectable IgG+. In some patients, antibodies were detectable in the first 3 days from disease onset.
Zhong *et al*.	China (347)	47 underwent molecular testing and were RT-PCR+.All 47 had detectable antibodies.
Zhao *et al*.	China (1)	Patient co-infected with HIV and HCV. Persistent negative RT-PCR (clinical diagnosis of COVID-19) and delayed antibody detectable at day 42
Okba *et al*.	Netherlands (3)	All the RT-PCR+ patients had detectable antibodies
Jin *et al*.	China (43)	All patients were RT-PCR+ and all had detectable antibodies up to 23 days from onset of symptoms
Liu *et al*.	China (214)	All 214 were RT-PCR+ and 146/214 had detectable IgM and 150 had detectable IgG. Antibodies were detectable within the first 6 days, peaked at around 10 days post symptom onset and declined after 35 days
Zhao *et al*.	China (173)	112/173 were RT-PCR+ and 161/173 had detectable IgG-IgM (143 IgM+ and 112 IgG+). A Total of 172 were RT-PCR+ or had detectable antibodies. There was no direct comparison between the tests
To *et al*.	Hong Kong (30)	23 patients were RT-PCR+ and 16 patients had paired RT-PCR and antibody data. Of the 16 with paired samples, 15 were IgG+ and 14 IgM+. Serum samples were collected and antibody tested from 14 days post symptom onset.
Xiao *et al*.	China (34)	All patients were RT-PCR+. All tested positive for IgM and IgG between 2-49 days with peak at 3 weeks.

* From medRixv and not certified by peer review; N, total number of participants in study (but not all underwent molecular and or serological testing)

**Rapid diagnostic tests for the detection of SARS-COV-2:** several rapid diagnostic tests (RDTs) have been developed and tested in single studies but none has been independently validated and commercially available for global use. Most of these tests have been developed and tested in Mainland China. Chen *et al*. described the development and testing of a lateral flow immunoassay (LFIA) that could detect SARS-COV-2-specific antibodies in serum within 10 minutes. This test detected antibodies in all 7 samples that were RT-PCR positive and 1 (out of 12) sample that was RT-PCR negative but clinically suspicious of SARS-COV-2 [38]. Yong *et al*. compared the diagnostic potential of RT-PCR and IgG/IgM antibody test for SARS-COV-2 and found that the RT-PCR was great at detecting early infection (92% of suspected COVID-19 cases (n=38) while antibody detection ranged from 23% (IgM) to 53% (IgG)) [42]. Antibody tests performed better at detecting prolonged infection (>7 days post symptom onset, ranging from 50% (IgM) to 92% (IgG). Li *et al*. evaluated rapid antibody test that could detect SARS-COV-2 antibodies in blood within 15 minutes and showed that 256 of 397 RT-PCR confirmed COVID-19 cases had detectable IgM-IgG antibodies [40]. Pan *et al*. used whole blood, serum and plasma samples to evaluate the rapid detection of SARS-COV-2 in RT-PCR confirmed samples and showed variation in agreement between the antibody test and RT-PCR ranging from 4-100% depending on the severity of the disease and timing of the test; the agreement was low within 7 days and highest around 14 days post onset of symptoms [39].

RDTs have also been developed and tested in other countries other than Mainland China. In Taiwan, Lee *et al*. tested 14 samples that were positive for RT-PCR using a rapid LFIA IgG/IgM antibody test kit which detected antibodies in 100% of the RT-PCR positive symptomatic patients and no antibodies in the RT-PCR positive asymptomatic patients and these antibodies were detected between 5 to 42 days post onset of symptoms [36]. In Italy, Spicuzza *et al*. compared 30 individuals, with 23 RT-PCR positive and 7 RT-PCR negative and showed that 19/23 were IgM/IgG positive (at day 9) using a 15-minute rapid test with whole blood, while only 1/7 RT-PCR negative was positive for the IgM/IgG at day 18 [35]. In Germany, Dohla *et al*. described an IgG/IgM-based rapid point-of-care (POI) test using blood drops that showed very low sensitivity of 36% and high specificity of 89% [37]. In Brazil, Castro *et al*. in a meta-analysis evaluating the different rapid antibody test kits and RT-PCR showed a 94% positive agreement between RT-PCR and serology tests where 120 participants tested positive for the IgG/IgM antibodies out of 128 who were RT-PCR positive [41].

Based on the above information, molecular testing by RT-PCR remain the main diagnostic test for early detection of SARS-COV-2 and antibody tests may play a secondary supportive role to indicate immune responses to the virus. However, if a rapid antibody test (or any other test type) with high sensitivity (and specificity) is developed and validated that could detect an active infection within minutes, this will have important implications in the early detection and treatment of patients with SARS-COV-2. The United States´ Food and Drug Administration (FDA) recently approved another molecular test (Xpert^®^ Xpress SARS-COV-2) developed by cepheid for emergency use only, which can produce results in less than an hour from sample collection [49]. Eventhough hands-on sample processing of swabs after collection is minimal, expensive equipment are still needed to perform the test, making it less suitable for mass screening in resource-limited settings (Table 2, Table 2 (suite)).

**Table 2(suite):** studies comparing the use of molecular and serology assays for the detection of SARS-CoV-2 with key findings from each study

Article	Location (N)	Summary of key findings
Haveri *et al*.	Finland (1)	Patient was RT-PCR+. IgM/IgG+ were detected 9 days post symptoms onset
Guo *et al*.	China (140)	82 patients were RT-PCR+ and 62/82 had detectable IgM antibodies.
Wolfel *et al*.	Germany (9)	All patients were RT-PCR+ and antibodies were detectable in at least 50% of the patients
*Liu *et al*.	China (179)	90 patients were RT-PCR+ and 77/90 had detectable antibodies
*Zhang P *et al*.	China (814)	122 patients were RT-PCR+, 660 RT-PCR- and 32 RT-PCR- but clinically confirmed as COVID-19. Of the 122 RT-PCR+, 106 had detectable IgG-IgM antibodies. Of the 32 Clinical COVID-19, 21 had detectable antibodies.
Zhang *et al*.	China (178)	Molecular test results were available for 31 patients who were all RT-PCR+. Only 16 of these 31 had antibody testing performed and antibodies were detectable in all 16 at day 5 (compared to no detection at day 0).
*Grzelak *et al*.	France (951)	Only 51 underwent molecular testing and were RT-PCR+. A total of 161 serum samples collected from the 51 patients’ overtime and 132 samples tested positive.
*Long *et al*.	China (285)	All 285 were RT-PCR+ and 262/285 had serum samples collected and antibodies were detectable in all of them.
Yong *et al*.	China (38)	All 38 were RT-PCR+. Antibodies (IgM, IgG or both) were detectable in 35 of the 38 patients.
**Studies comparing the use of molecular and rapid diagnostic serology assays for the detection of SARS-CoV-2 with key findings from each study**.		
Spicuzza *et al*.	Italy (37)	Only 30 underwent molecular testing and 23 patients were RT-PCR+. 19 of 23 had detectable antibodies by rapid test. Antibody tests were performed and detected 9-18days post symptoms
Pan *et al*.	China (105)	86 patients were RT-PCR+ and 67 patients had paired data for RT-PCR and antibodies. Antibodies were detectable in the first 7 days and peaked on average 15 days from symptoms onset
Li *et al*.	China (525)	397 patients were RT-PCR+. 256 of 397 had detectable IgM/IgG antibodies.
Dorla *et al*.	Germany (49)	All participants underwent molecular testing and 22 tested positive for qPCR. 11 of the 49 (8 qPCR+) had detectable antibodies.
#Castro *et al*.	Brazil (128)	In one of the tests evaluated, 128 patients were RT-PCR+. Of these, 120 tested positive for IgM, IgG or both. For other tests evaluated, RT-PCR and antibody tests were not directly compared by RT-PCR showed sensitivity of 95-100% while the total or individual antibody tests showed sensitivity of 56-100%
Chen *et al*.	China (19)	7 patients were RT-PCR+. All 7 had detectable antibodies
Lee *et al*.	Taiwan (14)	All patients were RT-PCR+ and 11/14 had detectable IgM and or IgG antibodies. Antibodies were detectable between 5-42 days post onset of symptoms.
Yong *et al*.	Singapore (28)	27 patients were RT-PCR+. All patients had detectable antibodies.

* From medRixv and not certified by peer review; #Meta-analyses; N, total number of participants in study (but not all underwent molecular and or serological testing)

**Genome sequencing of the SARS-COV-2 strains in Africa:** as of 21^st^ May 2020, there were 186 SARS-COV-2 genome sequences that have been completed in eight African countries (out of more than 30,000 sequences globally), as follows: Democratic Republic of Congo, DRC (124), Senegal (22), Ghana (14), South Africa (17), Algeria (3), Gambia (3), Egypt (2) and Nigeria (1) [50-52]. This suggests that though there is progress, from the first published sequenced in Nigeria on March 6, 2020 [52], Africa still needs to up its game and build more capacity with respect to genome sequencing. Understanding sequence distribution of the SARS-COV-2 strains circulating on the continent would help in understanding transmission dynamics and could eventually assist in the development of vaccine against COVID-19. The African sequences are relatively widely distributed within the global sequences [50]. This suggests importation of virus from different regions of the world. This may also suggest that effective vaccines that may be developed and tested in other parts of the world may also work well within Africa and there may be no need for continent-specific vaccines. However, vaccine testing in Africa is still strongly recommended.

**SARS-COV-2 sequencing and diagnosis (implications for Africa):** of all the studies on the various diagnostic and antibody detection tests identified and discussed in this review, none was conducted in Africa. This may be as a result of most African countries still largely focusing on early detection (by RT-PCR of SARS-COV-2) and treatment to control and prevent the pandemic taking hold in Africa. In addition, most of the ongoing research studies we are aware of on the African continent are focused on comparing treatment options and management of COVID-19 patients rather than evaluating diagnostic tests, as the RT-PCR is still the test of choice. There are reports that there are already a few LFIA tests developed in South Africa that are currently being tested, but results are not yet available [6]. The findings from the 2 case reports that COVID-19 clinically-diagnosed patients with HIV had persistent RT-PCR negative tests and delayed seroconversion from onset of symptoms might have clinical implications for the diagnosis and management of COVID-19 patients with HIV co-infection in Africa given that most African countries have high prevalence of HIV. These data however should be interpreted with caution. Africa is also contributing to the genome data with 8 countries so far (as of 21^st^ May 2020) reporting data on genome sequencing [50-52]. This is commendable but more countries and governments, with the support of international organisations such as the WHO, will need to support and encourage more collection and sequencing of the various circulating strains on the continent.

Institutions such as the pathogens genomics intelligence institute (PGII) of the Africa CDC will be instrumental in facilitating the sequencing of genomes for SARS-COV-2 and other pathogens and strengthening health systems and institutions for improved prevention, detection and response to public health threats on the continent and beyond [53]. COVID-19 pandemic has also presented the opportunity for African researchers in science and technology to collaborate more and pull resources together to increase research on COVID-19 and other infectious diseases in Africa [54]. For example, researchers may be able to share information or collaborate so that those with more resources could assist in sequencing the strains from countries with less resources. DRC has contributed more than 66% of the SARS-COV-2 genome sequences from Africa likely due to the established infrastructure from the Ebola virus response. Other African countries may leverage this available infrastructure to sequence more genomes.

Even though we performed a comprehensive search for articles to include in this review, the search was not exhaustive as we did not search all available databases. We are confident that even if we missed any studies, the evidence will not be drastically different from that presented in this review. Because RT-PCR was used as the gold-standard, we limited our selected articles to only studies that performed RT-PCR in addition to the serology test for comparison purposes and studies that performed RT-PCR alone or serology test alone were not considered.

## Conclusion

As SARS-COV-2 infections continue to rise on the continent, Africa will need to join the rest of the world in the development and testing of new diagnostic methods. Further research on the diagnosis of SARS-COV-2 in patients with infectious and non-communicable diseases (such as HIV, tuberculosis, diabetes) and other potential at-risk patient groups (such as pregnant women) is also needed to understand the relationship between comorbidities and COVID-19 pathogenesis as well as the effect of COVID-19 on the pathogenesis of other diseases and vice versa. In addition, more genome sequencing of African strains, building collaborations between African researchers and development of capacity to complete genome sequencing in most African countries should be encouraged and supported.

### What is known about this topic

- The challenges associated with the different diagnostic tests for SARS-COV-2, but limited data on direct comparisons of the different tests;
- Limited infrastructure for mass testing and sequencing of SARS-COV-2.

### What this study adds

- A detailed review of available evidence on genome sequencing in Africa, the challenges and the opportunities;
- A comprehensive synthesis of available evidence for the laboratory diagnosis of SARS-COV-2, direct comparisons between the performances of the different tests, research and development needs for the different tests in Africa, the challenges and the opportunities;
- Discussions and reflections on the state of genome sequencing and mass screening of SARS-COV-2 in Africa, and future directions.
